# Impaired endothelial function in pediatric patients with turner syndrome and healthy controls: a case-control study

**DOI:** 10.1186/1687-9856-2012-5

**Published:** 2012-04-02

**Authors:** Clodagh S O'Gorman, Catriona Syme, Tim Bradley, Jill Hamilton, Farid H Mahmud

**Affiliations:** 1 Divisions of Endocrinology, The Hospital for Sick Children Research Institute, University of Toronto, Toronto, Canada; 2 Divisions of Cardiology, The Hospital for Sick Children Research Institute, University of Toronto, Toronto, Canada; 3 Department of Pediatrics, The Hospital for Sick Children Research Institute, University of Toronto, Toronto, Canada; 4 Physiology and Experimental Medicine Program, The Hospital for Sick Children Research Institute, University of Toronto, Toronto, Canada; 5 Department of Paediatrics, Graduate Entry Medical School, University of Limerick, Limerick, Ireland

**Keywords:** Turner syndrome, Endothelial function, Adolescents, Pediatrics

## Abstract

**Background:**

Turner Syndrome women are at high risk of vascular disease and the assessment of early risk factors in Turner Syndrome girls is an emerging focus of research. Our objective was to evaluate endothelial function (EF), a preclinical measure of atherosclerosis, in Turner Syndrome girls compared with controls.

**Methods:**

A cross-sectional case-control study of Turner Syndrome girls and healthy controls. Subjects underwent fasting insulin and glucose with calculation of HOMA-IR, fasting lipid profile, anthropometrics, and EF testing using peripheral arterial tonometry (PAT). Subjects, aged 10-18 years, had karyotype-confirmed Turner Syndrome; growth hormone (GH), thyroxine and estrogen use were not exclusion criteria. Controls were age- and BMI-matched healthy girls. Fifteen Turner Syndrome and 15 controls were recruited.

**Results:**

Turner Syndrome girls had lower height, higher HDL and higher waist:height ratio than controls. PAT-hyperemia ratio (RH-PAT) scores were lower in Turner Syndrome (1.64 ± 0.34 vs. 2.08 ± 0.32, p = 0.002) indicating impaired EF. Among Turner Syndrome, RH-PAT did not vary with estrogen therapy or with karyotype 45,XO compared with other karyotypes. However, endothelial function was better in GH-treated compared with GH-untreated Turner Syndrome (1.80 ± 0.36 vs. 1.4 + 0.22, p = 0.02) although there were no differences in HOMA-IR, adiponectin or IGF-1.

**Conclusion:**

Girls with Turner Syndrome exhibit impaired endothelial function compared with controls, which may explain higher risk for vascular disease. GH may protect endothelial function in Turner Syndrome.

## Introduction

Turner Syndrome, a common genetic disorder affecting 1 in 2500 live-born females, is caused by complete or partial loss of × chromosome [1]. Despite significant advances in diagnosis and treatment in pediatric settings, Turner Syndrome patients experience high rates of cardiovascular disease such that adult females with Turner Syndrome have 3.47 and 2.21 standardized mortality ratios of coronary and cerebrovascular death respectively [2]. The majority of this excess mortality risk encompasses non-congenital circulatory disease [3]. However, this relates to underlying congenital structural and functional arterial abnormalities, which predispose these patients to aortic dilatation and aneurysm [4]. Additionally, Turner Syndrome patients exhibit a clustering of acquired cardiovascular disease risk factors, including increased rates of hypertension, glucose intolerance, obesity and dyslipidemia, which are also impacted by treatment regimens including growth hormone (GH) and estrogen [1].

Adolescents and young adults rarely experience cardiovascular events, and surrogate markers of cardiovascular disease are needed to evaluate asymptomatic patients. Impaired endothelial function is an initial step in the development of atherosclerosis, and represents an early and reversible step in the vascular disease process [5,6]. The measurement of endothelial function can be used as a surrogate marker to assess cardiovascular risk [7-10]. In prior clinical studies, peripheral arterial tonometry (PAT) testing has been used to measure endothelial function in both high risk adult [11] and pediatric populations [12,13]. Assessment of endothelial function has not previously been reported in pediatric Turner Syndrome populations and it is unclear whether Turner Syndrome is an independent risk factor for endothelial damage or if acquired cardiovascular risk factors impact endothelial function in this high risk patient group [14,15].

The objectives of this study were to evaluate endothelial function, as a marker of early vascular disease, in a group of pediatric Turner Syndrome girls in relation to an age-, sex- and body mass index (BMI)-matched healthy control population, and to assess the impact of disease-related treatment factors and acquired cardiovascular risk factors on endothelial function in the Turner Syndrome study group.

## Materials and methods

Institutional ethical approval was obtained for this case-controlled cross-sectional study. Inclusion criteria for Turner Syndrome subjects included: karyotype-confirmed diagnosis of Turner Syndrome; patients followed in our endocrinology clinic; and aged 10-18 years. Individuals receiving human growth hormone (GH), L-thyroxine or estrogen, or with a history of congenital heart disease, were eligible for inclusion. All patients taking L-thyroxine were on stable therapy with thyroid function within the normal range. Subjects took their medications as usual on the day of the study. Controls were healthy girls, age- and BMI-matched to TS subjects and recruited as part of previous studies using the same testing protocol. Exclusion criteria included: inability of the family and/or patient to comply with study protocol; previous diagnosis of Type 1 or Type 2 Diabetes Mellitus; taking medications for the treatment of disorders of glucose, insulin or lipid metabolism.

Each subject attended the Hospital for Sick Children for full evaluation on two separate days. On one day, subjects had fasting bloodwork including cholesterol profile (triglycerides, total cholesterol, high and low density lipoprotein cholesterol [HDL and LDL]), adiponectin, insulin and glucose. Homeostatic model assessment of insulin resistance (HOMA-IR) was calculated based on fasting insulin and glucose values: [16].

HOMA-IR=[Glucose (mmol/L)×Insulin (mU/L)]/22.5

Brachial arterial blood pressure (BP) (systolic, diastolic, and mean) and heart rate were recorded in the left arm using an automated Dinamap sphygmomanometer (Critikon Dinamap, Minneapolis, MN, USA). Each subject underwent history, examination and anthropometrics by a single examiner. Height, in metres (m), was measured using a wall-mounted stadiometer; and weight, in kilograms (kg), was assessed using calibrated electronic scales. BMI was calculated as kg/m^2^. Pubertal status was assessed by a single examiner according to the methods of Tanner [17].

On a different day, subjects returned fasting for endothelial function testing. PAT (Itamar Medical, Caesarea, Israel) is a non-invasive test which uses pneumatic probes, similar in shape to a thimble, which cover the fingertip and apply a uniform pressure field which allows for the measurement of the pulsatile oscillations of the digital vascular bed microcirculation. The PAT-hyperemia ratio (RH-PAT) shows a strong sensitivity and specificity for the detection of coronary endothelial function, which has been shown to predict cardiovascular events in adult patients [7,10]. PAT has been used previously as a surrogate marker of early endothelial function in paediatric populations [13,18] and, in adolescents it has been validated in a repeated measures study [19]. PAT probes are placed on finger II or III of each hand. After a 5 minute equilibration period, a BP cuff is inflated on the study arm 40 mmHg above systolic BP for 5 minutes. The cuff is then deflated and tonometric recording is completed for an additional 5 minutes. The RH-PAT was determined for each patient and each recording was analyzed individually. Lower RH-PAT index scores indicate worse endothelial function and increased risk for atherosclerosis.

Fifteen Turner Syndrome subjects were recruited, and matched for age and BMI with 15 healthy control girls (Table 1). All girls and their parents consented to participate. All subjects completed the protocol. For the girls with TS, karyotypes were as follows: 45,XO N = 6; 45,XO/46,XX mosaicism N = 1; 45,XO/47,XXX mosaicism N = 1; complex translocations N = 3; isochrome q material N = 2; 45,XO/46,XY mosaicism N = 2. Two subjects had aortic stenosis, including one with coarctaction repair several years previously. One additional subject had a bicuspid aortic valve. No other TS subjects had any known cardiovascular disease. Fourteen TS subjects were taking medications, including: N = 6 estrogen supplementation (N = 1 estrogen only; n = 1 estrogen and progesterone; N = 1 estrogen, progesterone and GH*; N = 3 oral contraceptive pill) and N = 7 GH supplementation (N = 4 GH only; N = 1 GH, estrogen and progesterone*; N = 1 GH and migraine prophylaxis; N = 1 GH with Ritalin and concerta) and N = 1 calcium supplementation (* denotes same patient).

**Table 1:** Baseline Demographics and results of investigations and PAT compared between girls with Turner Syndrome and Control girls

	Turner Syndrome N = 15	Control Subjects N = 15	p
Age	13.5 (2.4)	14.3 (1.7)	0.38
Systolic BP (mm Hg)	113.8 (12.2)	106.6 (5.5)	0.06
Diastolic BP (mm Hg)	65.8 (11.5)	70.18 (3.12)	0.21
Height (m)	1.42 (0.11)	1.64 (0.15)	**0.0019**
Weight (kg)	44.2 (13.0)	54.5 (15.5)	0.07
BMI (kg/m^2^)	21.5 (4.5)	20.8 (3.4)	0.64
Waist Circumference (cm)	73.1 (10.9)	71.4 (12.8)	0.71
Waist:Height Ratio	0.51 (0.06)	0.44 (0.08)	**0.0054**
Fasting glucose (mmol/L)	4.55 (0.91)	4.65 (0.47)	0.74
LDL cholesterol (mmol/L)	2.29 (0.58)	2.56 (0.73)	0.29
HDL cholesterol (mmol/L)	1.53 (0.51)	1.00 (0.32)	**0.003**
Total cholesterol (mmol/L)	4.27 (0.57)	4.08 (0.77)	0.46
Triglycerides (mmol/L)	1.01 (0.48)	0.95 (0.34)	0.71
HDLc:Triglycerides ratio	2.17(1.82)	1.26(0.70)	0.10
RH-PAT	1.64 (0.34)	2.08 (0.32)	**0.002**

Data are expressed as mean (SD)

### Biochemical analyses

Insulin was measured by chemiluminsecence using the Siemens Immulite 2500 (range of assay 15-2165 pmol/L, intra-and inter-assay coefficient of variation [CV] <7.6%). Adiponectin was measured by ELISA (Linco Research Inc., Range: 1-100 ng/mL; inter-assay CV 2.4 - 8.4%).

### Data analyses

All measures are expressed as mean value ± standard deviation (SD). Baseline characteristics were compared with paired t-tests (due to age- and BMI-matching of patients) and McNemar test. Associations between baseline characteristics and RH-PAT were quantified with Pearson Correlation Coefficients. Linear regression was used to determine whether baseline factors were correlated with outcome measures. All analyses were conducted with SAS, version 9.1 (SAS Institute Inc., Cary, NC).

## Results

There were no statistically significant differences between the Turner Syndrome and control groups for age, BP, weight or BMI. Control subjects were significantly taller than Turner Syndrome subjects. There was no difference in waist circumference between the groups; but, the waist:height ratio was higher in Turner Syndrome than in controls (Table 1). There were no differences between the groups for fasting glucose, LDL, total cholesterol, or triglycerides. HDL was higher in TS subjects.

The ages of GH-treated [N = 7] and GH-untreated [N = 8] Turner Syndrome girls were not different (12.7 ± 2.0 years versus 14.3 ± 2.4 years, t = -0.79, p = 0.22) and IGF-1 levels were not different between GH-treated and GH-untreated girls (423.8 ± 122.8 versus 336.7 ± 103.6 mcg/L respectively, p = 0.23). Among the GH-treated Turner Syndrome girls, GH treatment duration was 4.6 ± 1.1 years, with prescribed GH doses 0.35 mg/kg/week. There were no statistical differences in HOMA-IR, adiponectin, lipid measurements or blood glucose between GH-treated and GH-untreated Turner Syndrome girls (see Table 2).

**Table 2:** Comparison of metabolic parameters between TS subjects GH-treated versus GH-untreated

	GH-untreated N = 8	GH-treated N = 7	p-value
Age (years)	14.31(2.38)	12.66(2.00)	0.22
Height (m)	1.43(0.13)	1.40(0.10)	0.95
Weight (kg)	48.01(13.75)	39.79(11.53)	0.17
BMI (kg/m^2^)	23.02(4.72)	19.82(3.78)	0.24
RH-PAT	1.44(0.26)	1.86(0.28)	**0.045**
Systolic BP (mm Hg)	113.0(11.56)	114.71(13.70)	0.69
Diastolic BP (mm Hg)	64.88(6.49)	66.86(16.04)	0.65
Waist Circumference (cm)	76.19(11.90)	69.50(9.05)	0.29
Waist:Height Ratio	0.53(0.07)	0.49(0.04)	0.34
HOMA IR	1.90(1.64)	0.68(0.71)	0.06
Adiponectin (ng/mL)	16466(9186)	12995(4466)	0.53
IGF-1 (mcg/L)	423.8(122.8)	336.7(103.6)	0.43
Glucose (mmol/L)	4.79(1.20)	4.29(0.31)	0.95
Insulin (pmol/L)	61.25(44.38)	34.60(20.33)	0.24
HDL (mmol/L)	1.30(0.32)	1.80(0.57)	0.09
LDL (mmol/L)	2.49(0.66)	2.07(0.41)	0.24
Total Cholesterol (mmol/L)	4.27(0.66)	4.27(0.30)	0.78
Triglycerides (mmol/L)	1.14(0.58)	0.89(0.36)	0.33
HDL:Triglycerides Ratio	1.78(1.69)	2.56(2.00)	0.22

Data are expressed as mean(SD)

RH-PAT scores were significantly lower in Turner Syndrome than in control subjects (1.64 ± 0.34 versus 2.08 ± 0.32, p = 0.002). Among girls with Turner Syndrome, RH-PAT scores did not vary with estrogen replacement therapy (1.56 ± 0.30 with estrogen replacement [N = 6] versus 1.69 ± 0.37 without estrogen replacement [N = 9], p = 0.64) or between those with 45,XO karyotype [N = 6] and other karyotypes [N = 9] (1.60 ± 0.46 versus 1.67 ± 0.26 respectively, p = 0.56). However, RH-PAT scores were higher in girls receiving GH therapy than in those not receiving GH therapy (1.86 ± 0.28 versus 1.44 + 0.26 respectively, p = 0.045) and in Turner Syndrome girls receiving GH therapy, PAT scores were similar to control girls (1.86 ± 0.28 versus 2.08 ± 0.32 respectively, p = 0.14).

In Turner Syndrome subjects, RH-PAT score did not correlate with any measures of glucose, adiposity or anthropometrics. In control subjects, RH-PAT correlated positively with age and systolic BP, and negatively with LDL and HDL.

## Discussion

This study demonstrates that Turner Syndrome girls, compared with healthy age-, sex- and BMI-matched controls, have impaired endothelial function, an early, pre-clinical marker of vascular disease. We did not observe any significant correlations with acquired CVD risk factors present in our TS cohort, including lipid profile, BP or markers of insulin resistance.

The increased risk of cardiovascular complications in Turner Syndrome patients is complex and encompasses both congenital and acquired lesions. Recent data from the UK observed that non-congenital circulatory disease accounted for 41% of absolute excess mortality in Turner Syndrome women compared with only 8% for congenital anomalies [3]. Our study describes a young cohort of reasonably healthy Turner Syndrome patients with inherent impaired vascular function, of whom only 3/15 have evidence of some cardiac lesion. The Turner Syndrome patients are compared to BMI-matched healthy controls, because obesity measures do impair endothelial function, as we have previously described in adolescents using the same measure [13]. Previous reports evaluating vascular function in Turner Syndrome have shown carotid intima media thickening present in two adult studies, without evidence of abnormal endothelial function (brachial artery reactivity or pulse wave velocity) [20,21]. However, these studies were conducted in older Turner Syndrome patients with significant differences in BP and BMI compared with healthy controls.

We identified higher HDL concentrations in Turner Syndrome subjects compared with controls, and no effect of GH treatment on any measured cholesterol parameter was observed. These results are different with respect to previous studies that have shown higher total cholesterol in TS subjects independent of age, BMI and karyotype [22] and higher total cholesterol but lower HDL in older Turner Syndrome girls compared with controls [23].

Additionally, we identified a tendency towards a decrease in fasting insulin and HOMA-IR with GH therapy. Previous reports have been mixed. One study identified increased fasting insulin and glucose in TS girls on GH therapy [24] and another found no difference in fasting insulin prior to GH therapy in TS girls compared with controls, but found increased fasting insulin following 6-12 months of GH therapy [25]. Compared with controls, in two studies, TS girls on GH therapy had an exaggerated hyperinsulinaemic response to glucose load [24,26]. The suggestion of a protective effect of GH on insulin dynamics and endothelial function in TS girls in this report indicates that confirmatory studies are required, using various modalities to assess endothelial function and glucose-insulin dynamics in TS girls at various ages and stages of puberty.

We did observe that girls with Turner Syndrome who were receiving GH therapy had higher RH-PAT scores than girls with Turner Syndrome who were not receiving GH therapy, suggesting that GH may offer some endothelial protection in girls with Turner Syndrome. This was seen in a small number of patients, yet differences in endothelial function were not noted with estrogen replacement. Using other measures of endothelial or vascular function, previous studies have identified a protective effect of GH therapy. Flow-mediated dilatation improves in GH deficient adults following 3-18 months of GH therapy [27]. GH deficient adolescents had impaired flow-mediated dilatation, and GH induced a larger increase in blood flow in GH treated adolescents than in untreated or healthy controls [28]. Some studies have failed to identify improvements in vascular function following GH therapy, including a recent study of endothelial dysfunction in GH deficient children [29]. Both GH and sex steroids influence vascular function; GH improves cardiac contractility and vasculature tone [30] while estrogen promotes the release of nitric oxide thus resulting in vasodilation [31]. There are various mechanisms by which GH may improve endothelial function in girls with Turner Syndrome. Firstly, clinical studies suggest that GH may be beneficial in non-GH-deficient (non-Turner Syndrome) individuals with visceral obesity, by reducing abdominal fat and improving insulin sensitivity [32]. Secondly, studies suggest that the GH-IGF axis is profoundly altered in Turner Syndrome, specific alterations include a reduction in bioactive IGF-1 [33]. Finally, in studies of animal models of GH deficiency, GH treatment has resulted in increased anti-oxidant capacity and increased resistance to oxidative stressors [34,35].

Physiologically, inherent differences have been described as part of the vasculopathy associated with Turner Syndrome. The precise mechanisms underlying this are likely multi-factorial and related to endothelial injury; alterations in Turner Syndrome patients include abnormities of collagen [36] and impaired release of nitric oxide [37]. Imbalances in matrix metalloproteinases (MMP) activity necessary to maintain normal vessel structure has also been postulated to be present in TS patients [4]. These changes are important in promoting aortic dilation and aneurysm formation; they also likely act in synergy with acquired cardiovascular disease risk factors to promote accelerated atherogenesis in Turner Syndrome.

Several features of this study warrant further comment. This report is limited by the small number of patients studied and by the absence of significant correlations between RH-PAT and known traditional cardiovascular risk factors in the Turner Syndrome cohort. We did not observe any significant correlations with acquired CVD risk factors present in our cohort, including abnormal lipid profile, hypertension or insulin resistance. However, our relatively small study size and the lack of wide variation in these variables may account for this finding. We also used historical healthy controls who had undergone previous endothelial function testing using the same measure and protocol. While this did allow for matching on the basis of several confounding variables including age, sex and BMI, data relating to pubertal status or markers of insulin resistance were not available for comparison in the healthy control group. Nonetheless, given the nature of Turner Syndrome, with requirements for pubertal induction, it is unlikely that groups could be matched simultaneously for age and pubertal status [38]. Finally, we included 1 Turner Syndrome girl with a history of CoArctation repair. But this group of Turner Syndrome girls has not had cardiac MRI performed and, similar to previous studies [39], it is therefore possible that MRI would identify additional girls with structural heart defects. This study confirms that assessment of endothelial function using the PAT technique is well-tolerated and suitable for use in high risk, adolescent pediatric cohorts. However, like other tests of endothelial functionthat serve as surrogate markers of atherosclerosis, additional longitudinal data are required to evaluate any predictive value of these measures for cardiovascular events in adulthood.

Our findings suggest that that Turner Syndrome is an independent risk factor for impaired endothelial function at a young age, but GH therapy may be protective in the Turner Syndrome population. Considering this inherent vasculopathy, it is important to monitor and intervene in these patients to reduce the additional burden of acquired cardiovascular disease risk factors (hypertension, glucose intolerance, obesity and dyslipidemia), which are commonly observed in adolescent and adult Turner Syndrome patients. Regular screening is essential so that sub-clinical treatable conditions can be identified, with the ultimate goal of improving morbidity and mortality.

## Conclusions

We identified evidence of impaired endothelial function in pediatric Turner Syndrome subjects, compared with age- and BMI-matched female controls. We suggest that pediatricians need to be aware of increased risk of early atherosclerosis in pediatric age range Turner Syndrome patients, and to intervene with diet and lifestyle modifications and medications as needed. We also suggest that longitudinal studies are required to follow Turner Syndrome girls throughout the age spectrum into adulthood to identify and follow evidence of atherosclerosis.

## Abbreviations

PAT: Peripheral Arterial Tonometry; BMI: Body Mass Index; HOMA-IR: Homeostasis Model Assessment - Insulin Resistance; RH-PAT: PAT-hyperemia ratio; HDL: High Density Lipoprotein cholesterol; GH: Growth Hormone; IGF1: Insulin-Like Growth Factor 1; LDL: Low Density Lipoprotein; BP: Blood Pressure; Kg: kilograms; M: metres; CV: coefficient of variation; SD: Standard deviation.

## Competing interests

The authors declare that they have no competing interests.

## Authors' contributions

CSOG designed the study, collected the data, analysed the data, wrote the manuscript. CS co- collected data and co-analysed data. TB co-designed the study and co-analysed the data. JH participated in study design, data analysis and preparation of the manuscript. FHM conceived the study, designed the study, analysed the data and co-wrote the final manuscript. All authors read and approved the final manuscript.

**Figure 1:**
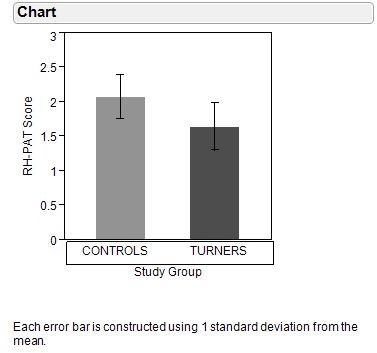
**Impaired endothelial function as shown as lower mean rh-pat score between subjects with turner syndrome and controls (mean + sd)**.
